# Characterization of NAD(P)H and FAD autofluorescence signatures in a Langendorff isolated-perfused rat heart model

**DOI:** 10.1364/BOE.9.004961

**Published:** 2018-09-21

**Authors:** João L. Lagarto, Benjamin T. Dyer, Clifford B. Talbot, Nicholas S. Peters, Paul M. W. French, Alexander R. Lyon, Chris Dunsby

**Affiliations:** 1 Photonics Group, Department of Physics, Imperial College London, Prince Consort Road, London SW7 2AZ, UK; 2 National Heart and Lung Institute, Imperial College London, Du Cane Road, London W12 0NN, UK; 3 Centre for Pathology, Imperial College London, Du Cane Road, London, W12 0NN, UK; 4 Authors contributed equally to this work; 5 Authors contributed equally to this work

## Abstract

Autofluorescence spectroscopy is a promising label-free approach to characterize biological samples with demonstrated potential to report structural and biochemical alterations in tissues in a number of clinical applications. We report a characterization of the ex vivo autofluorescence fingerprint of cardiac tissue, exploiting a Langendorff-perfused isolated rat heart model to induce physiological insults to the heart, with a view to understanding how metabolic alterations affect the autofluorescence signals. Changes in the autofluorescence intensity and lifetime signatures associated with reduced nicotinamide adenine dinucleotide (phosphate) (NAD(P)H) and flavin adenine dinucleotide (FAD) were characterized during oxygen- or glucose-depletion protocols. Results suggest that both NAD(P)H and FAD autofluorescence intensity and lifetime parameters are sensitive to changes in the metabolic state of the heart owing to oxygen deprivation. We also observed changes in NAD(P)H fluorescence intensity and FAD lifetime parameter on reperfusion of oxygen, which might provide information on reperfusion injury, and permanent tissue damage or changes to the tissue during recovery from oxygen deprivation. We found that changes in the autofluorescence signature following glucose-depletion are, in general, less pronounced, and most clearly visible in NAD(P)H related parameters. Overall, the results reported in this investigation can serve as baseline for future investigations of cardiac tissue involving autofluorescence measurements.

## 1. Introduction

Heart diseases including ischemia-reperfusion injury during acute myocardial infarction and chronic heart failure are characterized by complex functional and morphological alterations of the myocardium leading to structural and energetic dysfunction [1,2]. In recent years, advances in interventional and surgical techniques have allowed access to measurements of cardiac function using intracardiac electrodes, 3D echocardiography or pressure wires. However, none of these approaches directly evaluates the viability of the myocardium itself, but rather the gross and late impairment in electrical and mechanical activity at the level of the whole ventricular chamber. These macroscopic approaches, whilst offering new insights, generally do not provide accurate prognostic information or treatment targeting, which is now known to be best determined by myocardial structural and functional abnormalities and their inhomogeneity, and therefore more sophisticated tools are required. In particular, techniques employing a label-free method for in situ and real time characterization of cardiac tissue to identify the early alterations in the failing heart would represent a useful diagnostic advance to target early therapeutic intervention.

Autofluorescence has been increasingly exploited to report structural and functional characteristics of biological specimens including in isolated mitochondria [3], cell cultures [4–7] and in vivo measurements [8–12]. In particular, the autofluorescence characteristics of nicotinamide adenine dinucleotide (NADH) and nicotinamide adenine dinucleotide phosphate (NADPH) – which are biochemically distinct but spectroscopically identical and together are often referred to as NAD(P)H fluorescence – and flavin adenine dinucleotide (FAD) have been subject to extensive investigation to help study the metabolic mechanisms underlying many pathological conditions including in cancer [13,14], Alzheimer’s disease [15] or in cardiovascular diseases [16]. The autofluorescence signature of NADH in tissue is inherently complex due to different conformations of the molecule and the number of enzymes that it can bind to, e.g. malate dehydrogenase (MDH) and lactate dehydrogenase (LDH) [17–20]. NADH autofluorescence decay characteristics are commonly modelled with a bi-exponential function, where the short (τ_1_) and long (τ_2_) components refer to the free and protein-bound states of the molecule. However, this interpretation has been questioned recently [21,22] by work suggesting that the change in NAD(P)H lifetime observed is also affected by changes in NADPH fluorescence. In this paper we report the changes in the fluorescence decay parameters observed but avoid attributing these to specific biochemical changes.

The application of tissue autofluorescence to study of the heart physiology is still emerging, but reports to date demonstrated the potential of this technique to report structural and functional changes in the myocardium both ex vivo [16,23] and in vivo [24,25]. In cardiac tissue, the autofluorescence signal is strongly dominated by cellular autofluorescence, for which NAD(P)H and FAD are the major contributors [23], [26–28]. Other fluorophores such as collagens type I and III [29] and elastin [24,30] exist in cardiac tissue in lesser amounts and can also have a small contribution to the fluorescence signal. The relative proportions of these fluorophores in cardiac tissue are expected to change in disease, and hence these fluorophores are potential targets to measure if they reflect the energetic and structural health of the heart muscle. For example, the autofluorescence signal from collagen is expected to increase due to scar tissue proliferation following myocardial infarction and in heart failure [31,32]. At the same time, changes in the metabolic pathways are likely to occur as result of mitochondrial and energetic dysfunction, which will affect the relative proportions of NADH and NAD^+^ and, similarly, FADH_2_ and FAD [23,33,34]. While structural alterations are relatively straightforward to report, due to the high quantum yield of collagen and its long fluorescence lifetime, metabolic alterations can be challenging to interpret due to the multitude of metabolic pathways and fluorophore species involved.

In a previous study [25], we reported the application of time-resolved autofluorescence spectroscopy to characterize the in vivo autofluorescence signature of cardiac tissue in a 16 week myocardial infarction heart failure rat model. Although we were able to report structural changes in the myocardium due to large collagen proliferation, measurement of metabolic alterations was more challenging due to the overwhelming collagen signal. Building upon this preliminary work, we exploit the Langendorff-perfused isolated rat heart model to induce physiological insults that promote metabolic alterations in normal cardiac tissue and investigate the autofluorescence intensity and lifetime signatures. Specifically, we aim to characterize variations in the autofluorescence signal associated with NAD(P)H and FAD autofluorescence following impairment of myocardial function via oxygen and glucose depletion without interference from the strong collagen autofluorescence signal that is present in heart failure. These studies establish a baseline that will hopefully help the interpretation of future autofluorescence measurements of cardiac tissue.

## 2. Materials and methods

### 2.1 Langendorff preparation

**Fig. 1:**
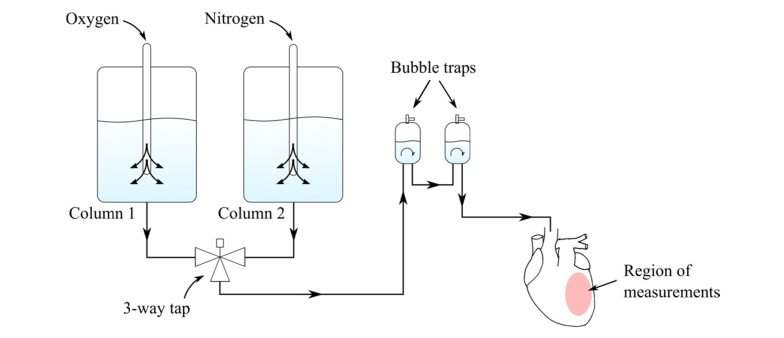
Schematic representation of the Langendorff experimental setup. A three-way tap was positioned immediately after the reservoirs to guarantee that only oxygenated or deoxygenation solution was delivered to the heart at a given time. Temperature of the heart was maintained at 37.5 ± 1 °C. Highlighted area in the left ventricular wall of the heart indicates the region of measurements. Black arrows indicate direction of the flow.

Adult male Sprague-Dawley rats (250–500 g) were anesthetized by administration of 5% isoflurane and sacrificed by cervical dislocation. Hearts were rapidly explanted and immersed in oxygenated iced Krebs-Henseleit (KH) solution. The composition of the KH solution was as follows (values in mM): 118 NaCl; 4.7 KCl; 0.94 MgSO_4_; 1 CaCl_2_; 1.2 KH_2_PO_4_; 25 NaHCO_3_; 11.5 C_6_H_12_O_6_. Hearts were cannulated through the aorta for retrograde perfusion with oxygenated (95%O_2_/5%CO_2_) KH solution in a Langendorff apparatus (130105EZ-V, AD Instruments, USA) within 5 minutes from explantation. After cannulation, hearts were allowed to stabilize for 10 minutes prior to recording autofluorescence and diffuse reflectance measurements. The flow rate was maintained at 18 ml/min throughout the entire procedure. Hearts with ventricular fibrillation, or with heart rate outside the range 200 – 400 bpm, were discarded. The experimental layout of the Langendorff system utilized in these experiments is illustrated in Fig. 1

. The apparatus comprises two columns containing solutions to be delivered to the heart by means of a peristaltic pump that maintained the pumping pressure close to physiological levels. Column 1 consisted of oxygenated KH solution. Column 2 was used to provide an insult-solution to the heart. Three different solutions were investigated: 1) oxygenated KH solution, which served as control (n = 4); 2) deoxygenated (95%N2/5%CO2) KH solution to induce a hypoxic state (n = 6); 3) glucose-depleted oxygenated solution to induce energy substrate privation (n = 5). In the latter solution, glucose was replaced by mannitol, a sugar alcohol with similar molecular weight to glucose that is nearly metabolically inert to mammals [35–38]. The solutions were combined using a three-way tap that allowed perfusion of the heart with only one solution at a given time. Two bubble traps were positioned in the circuit to prevent air bubbles from entering the heart. The set-up also included instrumentation to monitor and control the temperature of the solutions, which were set to maintain the temperature of the heart at 37.5 ± 1°C throughout the experiments.

### 2.2 Optical setup

The optical setup utilized in these measurements was described previously [25]. In brief, pulsed excitation light was provided by two laser diodes at 372 nm (LDH-P-C-375B, PicoQuant GmbH, Germany) and 438 nm (LDH-P-C-440B, PicoQuant GmbH, Germany) operating at 20 MHz. Excitation light was delivered to the sample via an optical fiber bundle consisting of three excitation fibers and fourteen collection fibers (NA = 0.22). Autofluorescence emanating from heart tissue was collected via the collection fibers and delivered to the detection system that provides spectral resolution by separating the autofluorescence signal into three spectral bands: 410 ± 10 nm (Channel 1), 455 ± 25 nm (Channel 2) and 525 ± 25 nm (Channel 3). Fluorescence in each spectral band was detected by a photon counting photomultiplier tube (PMT, PMC-100-1, Becker-Hickl GmbH, Germany). The three PMTs were connected to a router (HRT-41, Becker-Hickl GmbH, Germany) and a time-correlated single photon counting (TCSPC) card (SPC-830, Becker-Hickl GmbH, Germany). For 438 nm excitation, only the long wavelength detection band was used, referred to hereafter as Channel 4. The naming convention of the different channels is summarized in Table 1

**Table 1:** Summary of the different detection spectral channels.

Name	λ_ex_ (nm)	λ_em_ (nm)
Channel 1	372	410 ± 10
Channel 2	372	455 ± 25
Channel 3	372	525 ± 25
Channel 4	438	525 ± 25

.

To realize diffuse reflectance measurements, the instrument included a white-light source (HL-2000, Ocean Optics, USA) and a spectrometer (USB-2000 + , Ocean Optics, USA). The optical fiber bundle used for autofluorescence measurements also included a single optical fiber to deliver white light to the sample and a single optical fiber to collect the reflected light and deliver it to the spectrometer. The entire setup was controlled by a custom application written in LabVIEW (National Instruments, USA).

### 2.3 Autofluorescence and diffuse reflectance measurements

**Fig. 2:**
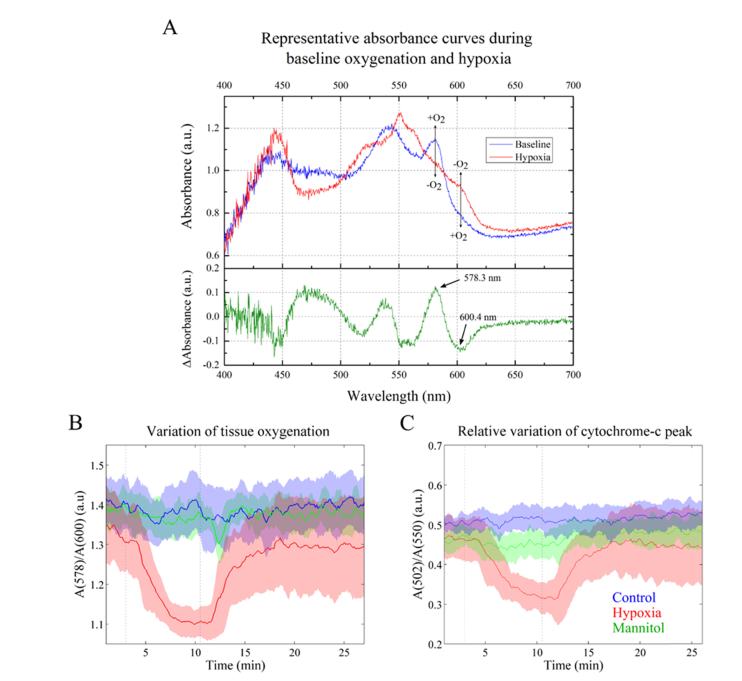
A) Representative absorbance spectra of oxygenated (blue curve) and hypoxic (red curve) cardiac tissue obtained from the Langendorff setup. The bottom curve (in green) shows the difference between the two spectra, i.e. A(baseline)-A(hypoxia). In the top panel, vertical black arrows indicate two wavelengths at which the differences between curves are maximized and the transition from + O_2_ to –O_2_ is also indicated. B) Estimated tissue oxygenation throughout the experiments calculated using the ratio of tissue absorption at 578 nm divided by the absorption at 600 nm. C) Estimated cytochrome c oxidation state estimated by the ratio of the tissue absorption at 502 nm divided by the absorption at 550 nm. Dashed vertical lines in grey indicate the switching time between oxygenated and deoxygenated Krebs solutions. Solid lines represent the mean taken over different hearts and shaded regions represent the region up to one standard deviation from the mean. Control, n = 4; hypoxia, n = 6; mannitol, n = 5.

Autofluorescence and diffuse reflectance measurements were recorded from the epicardial surface of the left ventricular anterior wall, as indicated in Fig. 1, by positioning the optical fiber probe tip in close proximity to the epicardial surface of the heart. The experimental protocol was divided in three phases, with a total data acquisition time of approximately 25.5 minutes: 1) 3 minutes of baseline measurements, during which the heart was perfused with oxygenated KH solution (Column 1, in Fig. 1); 2) 7.5 minutes of perfusion with oxygenated, deoxygenated or glucose-depleted KH solution (Column 2); 3) 15 minutes of reperfusion with oxygenated KH solution (Column 1).

Autofluorescence and diffuse reflectance data were acquired sequentially every 5 seconds. The integration times used in a single acquisition were 1 s for UV excitation, 1 second for blue excitation and 50 ms for diffuse reflectance measurements with white light illumination. For autofluorescence measurements, the output power at the sample was limited to a maximum of 25 μW for 372 nm excitation and 250 μW for 438 nm excitation and kept constant through the entire duration of the protocol.

### 2.4 Data analysis

#### 2.4.1 Diffuse reflectance data

Diffuse reflectance spectra from cardiac tissue were normalized to the diffuse reflectance signal from a white reference target (WS-1-SL, Labsphere, USA). Diffuse reflectance data are reported in units of absorbance calculated as:

$$A(\lambda )={\text{log}}_{10}\left(\frac{I(\lambda )}{I_{0}(\lambda )}\right)$$ (1)

where *A* is the tissue absorbance, *I* is the measured spectrum and *I_0_* is the calibrated spectrum of the white light source.

#### 2.4.2 Autofluorescence intensity data

Autofluorescence intensity data in each spectral channel of the time-resolved spectrofluorometer was normalized to the average of the first minute of baseline measurements. To estimate relative rates of glycolysis and oxidative phosphorylation, we calculated the optical redox ratio, RR, as the normalized autofluorescence intensity of NAD(P)H divided by that of FAD, at 372 nm excitation, which in our instrument is equivalent to the ratio of autofluorescence between Channels 2 and 3, i.e.

$$RR=\frac{I_{\text{CH}2}}{I_{\text{CH}2}+I_{\text{CH}3}}\approx \frac{\text{NAD}(P)H}{\text{NAD}(P)H+\text{FAD}}$$ (2)

#### 2.4.3 Autofluorescence lifetime data

Fluorescence signal from biological tissue emanates from multiple fluorescent species that give rise to complex fluorescence decay profiles and are typically described by a multi-exponential decay model. However, a common approach to simplify the analysis is to describe the fluorescence decays with a double exponential decay model, as shown in Eq. (3).

$$I(t)=a_{1}e^{\left(-t/\tau_{1}\right)}+a_{2}e^{\left(-t/\tau_{2}\right)}$$ (3)

where *τ*_1_, *τ*_2_ and *a*_1_, *a*_2_ refer to the fluorescence lifetimes and pre-exponential factors of the fast and slow components in the decay, respectively. The intensity weighted mean lifetime *τ*_mean_ can be calculated as follows:

$$\tau_{\text{mean}}=\frac{a_{1}\tau_{1}^{2}+a_{2}\tau_{2}^{2}}{a_{1}\tau_{1}+a_{1}\tau_{2}}$$ (4)

The fractional contribution of the fast decay component to the initial fluorescence signal, *α*_1_, was calculated as shown in Eq. (5).

$$\alpha_{1}=\frac{a_{1}}{a_{1}+a_{2}}$$ (5)

Fluorescence lifetime data were analyzed using a non-linear least squares fitting routine from MATLAB (*lsqnonlin* function, R2011b, The Mathworks, Inc., USA) to minimize the goodness of fit chi-square value, χ^2^

$$\chi^{2}=\sum_{k=1}^{n}\frac{{\left(I(t_{k})-I_{\text{model}}(t_{k})\right)}^{2}}{I(t_{k})}$$ (6)

where *n* is the number of bins in the histogram (n = 1024) and *I*_model_ is the model decay function convolved with the instrument response function (IRF). The fitting model included the double exponential decay model described by Eq. (3), the measured background offset, the afterpulsing probability of the detectors and incomplete decay estimation. The IRF for each detection channel was measured using reference fluorophores with known decay characteristics: DAPI (Sigma-Aldrich, USA) for 372 nm excitation in all detection channels; and Erythrosin B (Sigma-Aldrich, USA) using 438 nm excitation. These reference dyes have lifetimes of approximately 200 ps, which are comparable to the pulse lengths of the lasers used and the transit time of the detectors.

## 3. Results

### 3.1 Diffuse reflectance measurements

Diffuse reflectance measurements can be used to report myoglobin and hemoglobin concentration [39], oxygen saturation [40] and oxidative state of tissue [41]. The diffuse reflectance spectrum of heart tissue heavily depends on the relative contribution of the chromophores present in the tissue. In cardiac tissue, the dominant chromophores are myoglobin and hemoglobin. However, the Langendorff set-up is blood-free and therefore the contribution of hemoglobin to the diffuse reflectance spectra is negligible. Figure 2(A)

shows representative absorption curves of well oxygenated and oxygen-depleted tissue, retrieved from baseline control and hypoxia measurements, respectively. The curve from well-oxygenated tissue (in blue) shows a characteristic myoglobin double peak profile in the 500 - 600 nm band, with local maxima at ~544 and ~578 nm, which is in agreement with previously reported values [42]. In contrast, the curve from oxygen-depleted tissue (in red) presents a single peak at around 550 nm, which is considerably shifted relative to values reported in literature, i.e. ~560 nm. While we observe a shoulder at ~560 nm that we can attribute to deoxygenated myoglobin, the peak at 550 nm is consistent with the absorption peak of reduced cytochrome c [43].

Given our interest in understanding the autofluorescence signatures of cardiac tissue during hypoxia, monitoring tissue oxygenation is critical to ensure that measurements of control and glucose-depleted samples are not impacted by variations in oxygen delivery/consumption. Typically, oxygenation can be reported using tissue absorbance values at key wavelengths in the red and infra-red regions (e.g. 660 nm and 950 nm), where the differences between oxygenated and deoxygenated myoglobin/hemoglobin curves are maximized [44]. In our case, since we are working in the visible spectrum, we calculated the difference between oxygenated and deoxygenated curves in the 400 – 700 nm band to find the wavelengths at which the difference is maximized (Fig. 2(A), bottom curve in green).

The difference between the oxygenated and deoxygenated curves is maximized at ~578 nm and ~600 nm. Thus, the absorbance ratio at these wavelengths provides an estimation of myoglobin oxygenation, and therefore tissue oxygenation, throughout the experimental protocol. Figure 2(B) shows the average oxygenation level – calculated as described above – for control, hypoxia and glucose-depleted (mannitol) experiments. As expected, tissue oxygenation decreased in hypoxia experiments (red curve) when perfusion was switched from oxygenated to deoxygenated solutions, returning to the baseline levels upon re-oxygenation. The curves for control and mannitol experiments are relatively flat, thus indicating that tissue oxygenation was approximately constant during measurements. This indicates that any variations in tissue autofluorescence during the control or mannitol experiments cannot be attributed to variations of tissue oxygenation. The hypoxia curve (in red, Fig. 2(B)) also indicates that changes in tissue oxygenation are only observed approximately two minutes after switching the perfusion solutions, which is consistent with the measured time required for the solution in each column to reach the heart.

Due to the clear presence of the reduced cytochrome c absorption peak in the oxygen depleted tissue at 550 nm, we calculated a second ratio to estimate the cytochrome c oxidation state. For this we chose a wavelength where there is no change in absorption with oxygenation (502 nm) and divided the absorption at 502 nm by the absorption at 550 nm. The results are shown in Fig. 2(C) and are consistent with those for the myoglobin oxygenation shown in Fig. 2(B).

### 3.2 Autofluorescence intensity measurements

**Fig. 3:**
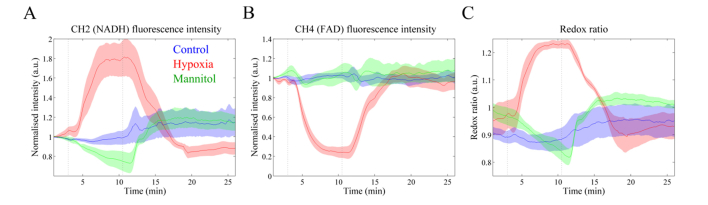
Variation of autofluorescence intensity signal in channels (A) 2 and (B) 4, corresponding primarily to NAD(P)H and FAD autofluorescence, respectively. (C) Redox ratio curves calculated according to Eq. (2). Solid lines represent the mean taken over different heart and shaded regions represent up to one standard deviation from the mean. Greyed dashed lines indicate the time when the perfusion solution was changed.

Cardiac tissue autofluorescence in healthy hearts emanates predominantly from NAD(P)H and FAD. Figure 3

shows the autofluorescence intensity measured in channels 2 and 4, which correspond primarily to NAD(P)H and FAD autofluorescence, respectively. In the hearts subjected to hypoxia, we observe an increase in NAD(P)H autofluorescence intensity upon perfusion with the deoxygenated KH solution (Fig. 3(A), red curve) and a similarly shaped decrease in FAD autofluorescence intensity (Fig. 3(B), red curve) until a stabilization plateau is reached. These observations result in an increase in the optical redox ratio during hypoxia (Fig. 3(C), red curve). The autofluorescence intensities of NADH and FAD return to baseline following reperfusion with oxygenated solution, albeit at a slower rate than they initially increased/decreased after perfusion with deoxygenated solution.

In hearts subjected to glucose depletion, we observed a decrease in NAD(P)H autofluorescence intensity (Fig. 3(A), green curve). Following the switch to glucose-free medium, NAD(P)H autofluorescence intensity continuously decreased with time and decreased more slowly than during hypoxia. No plateau was observed within the 7.5 minutes insult. We found an inconsistent response in FAD autofluorescence intensity in Channel 4: 1 out of 4 specimens showed an increase in autofluorescence during the insult; in the remaining 3 specimens the autofluorescence signal persisted relatively unchanged throughout the course of the experiments. This resulted in a relatively flat mean autofluorescence intensity over time (Fig. 3(B), green curve).

### 3.3 Autofluorescence lifetime measurements

#### 3.3.1 Channel 2 (Ex. 372 nm, Em. 430 - 480 nm): NAD(P)H

Figure 4

**Fig. 4:**
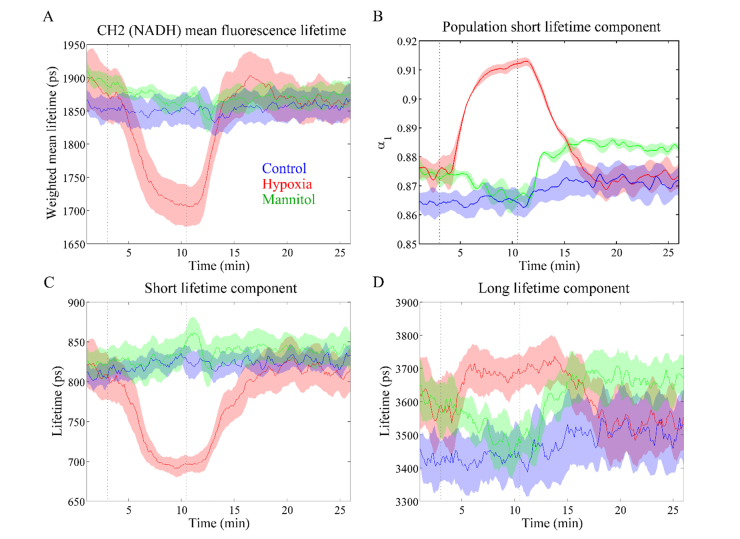
Autofluorescence lifetime parameters measured in channel 2: (A) mean lifetime; (B) contribution of short lifetime component *α*_1_; (C) short lifetime *τ*_1_; (D) long lifetime *τ*_2_. NADH is the major fluorophore contributing to the autofluorescence signal in this channel. Decrease of short component lifetime with concomitant increase in its contribution suggests increase in free-NADH content during hypoxia. Solid lines represent the mean taken over different heart and shaded regions represent up to one standard deviation from the mean. Greyed dashed lines indicate the time when the perfusion solution was changed.

shows the autofluorescence lifetime signature of cardiac tissue in detection channel 2. The autofluorescence signal measured in detection channel 2 emanates predominantly from NAD(P)H, given the large overlap between NAD(P)H autofluorescence emission and the spectral band of this channel. If we average the measurements taken during the whole course of control experiments, we calculate a weighted mean lifetime (*τ*_mean_) of 1.85 ± 0.04 ns, a short lifetime (*τ*_1_) of 0.82 ± 0.02 ns, a long lifetime (*τ*_2_) of 3.47 ± 0.16 ns and a fraction of the short lifetime component (*α*_1_) of 0.87 ± 0.02 (n = 4). The low standard deviations are indicative of the low variation in the autofluorescence decay characteristics of the tissue throughout the course of control measurements.

We observe a decrease in the mean autofluorescence lifetime of cardiac tissue during hypoxia (Fig. 4(A), red curve), which can be traced back to a decrease in short lifetime component τ_1_ (Fig. 4(C)) and increases in the long lifetime component τ_2_ (Fig. 4(D)) and in the population associated with the short lifetime component α_1_ (Fig. 4(B)).

Similarly to the steady-state autofluorescence data, changes in autofluorescence decay characteristics are less pronounced during perfusion with mannitol and concomitant glucose starvation than during hypoxia. Indeed, our results suggest that glucose deprivation does not induce alterations in the mean autofluorescence lifetime of NAD(P)H at this detection wavelength. Clear changes are only observed in the long lifetime component, which shows a slight decrease upon perfusion with mannitol, returning to the baseline upon reperfusion with glucose. We also note a slight increase in the contribution of the short lifetime component to the autofluorescence decay after reperfusion with glucose-based solution. As explained above, it is possible that changes occurring after reperfusion are related to increased diastolic pressure due to loss of myocardial function during glucose deprivation. Overall, these results suggest that detection channel 2 is less sensitive to changes induced by glucose deprivation compared to channel 3 (see below).

#### 3.3.2 Channel 3 (Ex. 372 nm, Em. 500 - 550 nm): NAD(P)H and FAD

Figure 5

**Fig. 5:**
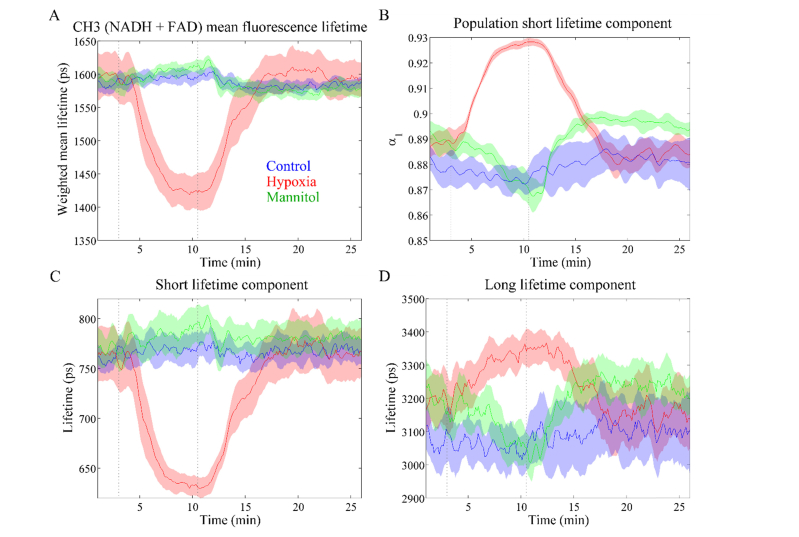
Autofluorescence lifetime parameters measured in channel 3: (A) mean lifetime; (B) contribution of short lifetime component *α*_1_; (C) short lifetime *τ*_1_; (D) long lifetime *τ*_2_. The autofluorescence signal in this channel emanates primarily from NADH and, to a lesser extent, FAD. Solid lines represent the mean taken over different heart and shaded regions represent up to one standard deviation from the mean. Greyed dashed lines indicate the time when the perfusion solution was changed.

presents the autofluorescence lifetime data for channel 3. The fluorescence lifetime patterns measured for this channel are similar to those of channel 2, particularly for hypoxia. This is an expected result, since the autofluorescence signal measured in this channel is dominated by NAD(P)H with a small contribution of FAD. With respect to control hearts, the average values of each parameter are as follows: *τ*_mean_ = 1.59 ± 0.01 ns; *τ*_1_ = 0.77 ± 0.02 ns; *τ*_2_ = 3.08 ± 0.15 ns; *α*_1_ = 0.88 ± 0.02 (n = 4). The mean autofluorescence lifetime measured in this channel is shorter than that of channel 2, suggesting decreased contribution of NAD(P)H with concomitant increase of FAD autofluorescence, given the shorter autofluorescence lifetime measured for channel 4, where FAD is believed to be the dominant fluorophore with negligible or no contribution from NAD(P)H, see the subsection that follows reporting the measurements for Channel 4.

Alterations in the autofluorescence signature of cardiac tissue induced by glucose deprivation are more clearly observed in detection channel 3 than in channel 2, although they are not as pronounced as changes induced by hypoxia. As in channel 2, we observe a decrease in the long lifetime upon perfusion with mannitol and consequent depletion of energy substrate (Fig. 5(D), green curve). More interestingly, changes in the contribution of the short lifetime component are more evident in this channel: it decreases with glucose deprivation and slowly returns to the baseline values when glucose is restored to the system (see Fig. 5(B)). The larger decrease in the contribution of the short component *α*_1_ can be explained by the decrease in the NAD(P)H fluorescence intensity (Fig. 3(A), green curve) relative to that of FAD, which is relatively constant (Fig. 3(B), green curve) and the shorter mean lifetime of FAD compared to that of NAD(P)H.

#### 3.3.3 Channel 4 (Ex. 438 nm, Em. 500 - 550 nm): FAD

**Fig. 6:**
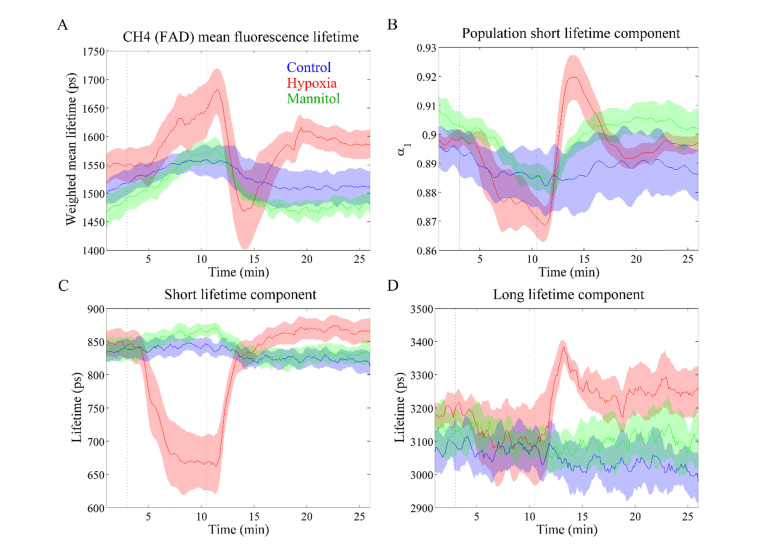
Autofluorescence lifetime parameters measured in channel 4: (A) mean lifetime; (B) contribution of short lifetime component *α*_1_; (C) short lifetime *τ*_1_; (D) long lifetime *τ*_2_. The major contributor to the autofluorescence signal in this channel is FAD. Solid lines represent the mean taken over different heart and shaded regions represent up to one standard deviation from the mean. Greyed dashed lines indicate the time when the perfusion solution was changed.

The autofluorescence decay signatures of heart tissue excited at 438 nm are presented in Fig. 6

. Changes in this channel are more complex than those previously described for detection channels 2 and 3. We observed a variation of ~50 ps in the mean autofluorescence lifetime of control specimens and also in the baseline measurements across hypoxia, mannitol and controls protocols that are possibly due to the fluorophores contributing to this channel being more sensitive to variations in e.g. the temperature of the perfusion solution or metabolic status. If we average all measurements for control specimens (n = 4), we obtain the following values: *τ*_mean_ = 1.52 ± 0.05 ns; *τ*_1_ = 0.83 ± 0.02 ns; *τ*_2_ = 3.05 ± 0.11 ns; *α*_1_ = 0.89 ± 0.02.

The autofluorescence signal measured in this channel exhibits a complex signature during hypoxia. The mean lifetime initially increases during oxygen deprivation, due to decreases in *τ*_1_ and *α*_1_. *τ*_2_ is slightly decreased during oxygen deprivation. Upon reperfusion with oxygenated solution, there is a rapid decrease in mean lifetime with an undershoot relative to baseline. Finally, the mean lifetime increases again to stabilise slightly above the baseline level. Changes in the average fluorescence lifetime after reperfusion can be traced back to rapid increases in the long lifetime (Fig. 6(D)) and contribution of the short lifetime component (Fig. 6(B)). We do not observe a similar pattern in any other autofluorescence parameter. Indeed, the results above show that changes in the steady-state autofluorescence signal occur at a slower rate in reperfusion relative to the initial oxygen depletion (see Fig. 3) and a similar pattern can be observed for the time-resolved parameters of channels 2 and 3. In contrast with NAD(P)H, FAD autofluorescence derives from mitochondria only [43]. Therefore, these alterations may also indicate a rapid reestablishment of the oxidative pathway. This would not be observed in the NAD(P)H autofluorescence properties since NADH is present in both the aerobic and anaerobic pathways. It is also interesting to observe that both lifetime components decrease during hypoxia, despite the increase in mean lifetime. This decrease is driven by the decrease in the contribution of the short lifetime component to the fluorescence decay. This component is commonly attributed to protein-bound FAD and thus these alterations suggest an increase in free FAD content during hypoxia.

Our results suggest that there is not much difference between control and mannitol groups. Although the average lifetime (see Fig. 6(A)) appears to increase after perfusion with mannitol, this was already increasing during baseline and, in general, the pattern is similar to that of the control. There is a slight decrease in the contribution of the short lifetime component during glucose deprivation. Overall, FAD autofluorescence reported in Channel 4 appears to be less sensitive to metabolic alterations due to glucose starvation than NAD(P)H autofluorescence (see Fig. 3(B)), as seen in Channels 2 and 3.

## 4. Discussion

In a previous study, we characterized the in vivo autofluorescence signature of cardiac tissue in a 16-week myocardial infarction heart failure model, where we were able to report changes in the autofluorescence signal due to major structural remodeling following collagen proliferation. While structural alterations were relatively straightforward to measure, changes in the autofluorescence properties associated to the metabolic signals of NAD(P)H and FAD were significantly more challenging to unravel, due to the overwhelming collagen signal and its characteristic long autofluorescence lifetime. To characterize the autofluorescence signatures of NAD(P)H and FAD without interference from collagen autofluorescence, in this study we measured the autofluorescence signals emanating from cardiac tissue in an isolated-perfused rat heart model. The Langendorff rat heart preparation is a well-characterized model that facilitates studies of myocardial physiology and is a convenient experimental preparation for optical measurements requiring controlled metabolic impairment of the myocardium. However, in healthy adult living hearts, the fuel that sustains cardiac cells derives from oxidation of fatty acids, which accounts for nearly 70% of the total energy production in the myocardium, while glycolysis plays a less relevant role [2]. In contrast, in the standard Langendorff preparation, glucose is provided in the perfusion solution as the energy substrate. Overall, great care is required when interpreting the results from the Langendorff model if they are to be extrapolated to the in vivo scenario, given the considerable metabolic shift between the two. In the future, it would be useful to perform further experiments to study the changes in autofluorescence seen in hearts perfused with a solution containing fatty acids instead of glucose, and also how a heart would respond to cessation of fatty acids supply. It would be interesting to study the effects of inhibiting oxidative phosphorylation through hypoxia and the cessation of glycolysis through a suitable metabolic modulator, e.g. 2-deoxyglucose, in order to investigate whether these changes are reflected in the optical parameters measured and the relative contributions of these two processes.

The Langendorff heart preparation is a technically challenging procedure that requires fine surgical skills to prepare the specimen and precise instrumentation control to balance physiological parameters during the protocol, such as perfusion pressure, temperature, oxygenation and salt concentration that are essential to maintain viable cardiac activity. Although cardiac activity can be maintained for several hours in a Langendorff protocol with adequate physiological conditions [45], myocardial function is slowly but progressively impaired. Thus, we chose to realize a short protocol (~30 minutes) during which myocardial function remains relatively unaltered, as verified by the stable heart rate throughout control measurements (data not shown) and stability of our control data.

A common concern associated with optical measurements in the heart is the natural movement that can produce artefacts in the measured data due to variable probe-to-target distance. In our measurements, the heart rate was maintained between 200 - 400 bpm, which corresponds to approximately 3 to 6 heart beats in each 1 s acquisition. This means that our autofluorescence measurements integrate signal from multiple cardiac cycles, thereby averaging the effects caused by the movement of the beating heart. Furthermore, autofluorescence lifetime measurements are inherently ratiometric and thus relatively insensitive to intensity excitation-collection geometry.

Optical measurements are practical in a Langendorff set-up since this is a blood-free model, which makes the contribution from hemoglobin absorption negligible. Thus the dominant absorber in the blood-free cardiac tissue is myoglobin. Myoglobin occurs at a lower concentration than hemoglobin in blood and presents similar spectral features to those of hemoglobin, only slightly red-shifted. As expected, our diffuse reflectance data show characteristic myoglobin features during perfusion with oxygenated solution. However, in hypoxia, we observe a single peak profile that is not consistent with the deoxygenated myoglobin spectral features and that we attribute to reduced cytochrome c. Cytochrome c is a key element in the electron transport chain of oxidative phosphorylation, being reduced by complex III and oxidized by complex IV [43]. In anaerobic conditions the ratio of reduced to oxidized cytochrome c increases, and thus its absorbance spectral features become more pronounced (single peak at 550 nm).

Autofluorescence in normal heart tissue is dominated by NAD(P)H and FAD. We note that free riboflavin and flavin mononucleotide (FMN) also contribute to the autofluorescence signal excited at 438 nm. However, results from previous studies showing that FAD comprises 84% [46] and ~60% [47] of total riboflavin in heart tissue suggest that fluorescence from FAD should dominate. Collagen and elastin that are also present as key structural components of the vascular network and extracellular matrix may also make a small contribution to the autofluorescence signal, particularly for UV excitation. In the emission band typically associated to collagen fluorescence, i.e. 400 - 420 nm (Channel 1), we measured low fluorescence intensities compared to other detection channels, suggesting that collagen autofluorescence in normal cardiac tissue is minimal. Furthermore, we note that the autofluorescence lifetimes measured in Channel 1 (~1.75 ns) are similar to those measured in Channels 2 and 3 and significantly shorter than the autofluorescence lifetimes of collagen and elastin [48,49]. We also measured the autofluorescence signature of a decellularized heart at 405 nm, 450 nm and 525 nm (excited at 375 nm) and extracted lifetimes of 6.1, 5.8 and 5.9 ns, respectively. These results further suggest that the autofluorescence signal measured from cardiac tissue predominantly emanates from NAD(P)H and FAD. In the future, it would be interesting to incorporate a spectrometer capable of recording the fluorescence emission spectra with higher spectral resolution than the three detection bands employed here. This could enable the positions of the NAD(P)H fluorescence emission peaks to be quantified more accurately and may provide additional information on its binding partners due to small spectral shifts observed when it binds to proteins [17–20].

We previously reported the autofluorescence signature of cardiac tissue in vivo, in a 16-week myocardial infarction heart failure model in rats [25]. Interestingly, the autofluorescence lifetimes measured in vivo are slightly shorter relative to those measured ex vivo, see Table 2

**Table 2:** Comparison of weighted mean autofluorescence lifetimes previously reported for healthy hearts in vivo [25] and in this study. In vivo measurements were realized in the anterior wall of the left ventricle.

	*τ*_mean_(ns)	
	*In vivo* [25]	Langendorff [this study]
Channel 1	1.56 ± 0.19	1.75 ± 0.13
Channel 2	1.69 ± 0.10	1.85 ± 0.04
Channel 3	1.39 ± 0.08	1.59 ± 0.01
Channel 4	1.35 ± 0.11	1.59 ± 0.01

. These differences potentially reflect the considerable metabolic shift from in vivo to ex vivo.

Depletion of oxygen or glucose considerably altered the autofluorescence signature of the myocardium. These alterations are observed in both intensity and lifetime parameters, although the latter are less pronounced. With respect to autofluorescence intensity measurements during hypoxia, our results are in close agreement with those recently published by others [23,50] in ischemia reperfusion Langendorff heart models. We observed an increase in NAD(P)H autofluorescence with concomitant decrease in FAD, which is consistent with increased glycolytic rate due to impairment of the mitochondrial function. Changes in autofluorescence intensity during hypoxia occurred predominantly in the first ~3 minutes of oxygen deprivation, after which the signal appears to stabilize. It is possible that, at this point, the oxidative metabolic pathway is completely inhibited and thus ATP for cardiac cells is synthesized only in glycolysis, which can occur in anaerobic conditions. Since glycolysis has substantially lower energetic yield compared to oxidative phosphorylation, the heart cannot be sustained exclusively by this pathway, which could explain the decrease in heart rate to approximately 200 bpm measured during hypoxia (data not shown). Maintenance of this pathway for a longer period would eventually result in irreversible cell damage and death. It is also interesting that changes in the autofluorescence intensity following reperfusion with oxygenated solution occurred at a slower rate (~5 min, from 10 to 90%) than changes upon perfusion with deoxygenated solution (~2 min, from 10 to 90%). The reasons behind this difference are unclear, although exposure to hypoxia may have caused impairment of cellular function in the viable myocardium and thus slow down the mechanisms to recover normal activity. Similar patterns have been observed in ischemia reperfusion models with respect to the diastolic pressure of the heart, which remains elevated after reperfusion [51] and may help explain our results.

Changes in the autofluorescence intensity during hypoxia were accompanied by changes in the autofluorescence lifetime parameters in all detection channels of our instrument. In channels associated to NAD(P)H autofluorescence (i.e. Channels 2 and 3), we observed a decrease in the mean lifetime that is essentially driven by a decrease in the short lifetime component and increase in its contribution to the overall fluorescence decay. The autofluorescence lifetime characteristics of Channel 2 correlate well with those measured in Channel 3, suggesting that similar fluorophore species contribute to the fluorescence decay in both channels, as expected. Indeed, considering the excitation and emission spectra of NAD(P)H and FAD, we expect NAD(P)H to contribute more significantly to Channel 3 autofluorescence compared to FAD. However, we note a decrease in the mean lifetime of Channel 3 relative to that of Channel 2, which suggests that FAD also has a relevant contribution to the autofluorescence signal in this channel. In Channel 4 however we expect the contribution from NAD(P)H to be negligible and the autofluorescence signal from FAD can be decoupled from that of NAD(P)H. During hypoxia we observe an increase in the mean lifetime of Channel 4, despite decreases in both the short and long lifetime components, through a corresponding decrease in the contribution of the short component to the decay (see Fig. 6). Interestingly, after reperfusion with oxygenated solution we measured a rapid shift with overshoot relative to baseline values in the *α*_1_ and *τ*_2_ parameters (see Fig. 6(B) and 6(D)). These alterations may indicate a rapid re-establishment of the oxidative pathway following reperfusion with oxygenated solution, but further studies are necessary to unravel the biochemical origin of this signal.

The autofluorescence signatures of cardiac tissue during glucose-depletion studies are more challenging to interpret as variations in both intensity and lifetime were less pronounced than those observed for hypoxia. As discussed previously, in the Langendorff preparation, glucose is provided as the energy substrate via the perfusion solution. Thus, when glucose is replaced by a metabolically inert compound such as mannitol in the perfusion solution, the substrate consumed to produce energy is not replenished, eventually leading to mitochondrial dysfunction. Accordingly, the levels of ATP in tissue are expected to decrease, contributing to progressive myocardial cell death and consequent impaired contractile function [52]. Changes in the autofluorescence decay parameters are less pronounced than those observed for hypoxia, which suggests slow but progressive ATP depletion. It is possible that small amounts of energy substrate were stored in heart cells and consumed when there was no other source of energy available. In general, the autofluorescence intensity parameters appear to be more sensitive to glucose starvation than the decay parameters. Changes in the autofluorescence lifetime parameters are minor and mostly clearly visible in the long lifetime component *τ*_2_ and in the contribution of the short lifetime component *α*_1_ of channel 3 (see Fig. 5).

Table 3

**Table 3:** Summary of variation of autofluorescence intensity and lifetime parameters during hypoxia and glucose-depletion measurements. Arrows indicate increase (↑) or decrease (↓) in the corresponding parameter relative to baseline measurements.

		Channel 2				Channel 4			
Excitation wavelength		372 nm				438 nm			
Detection wavelength		430 – 480 nm				500 – 550 nm			
Dominant fluorophore		NAD(P)H				FAD			
Protocol		Hypoxia		Glucose-depletion		Hypoxia		Glucose-depletion	
		Insult	Reperfusion	Insult	Reperfusion	Insult	Reperfusion	Insult	Reperfusion
Fluorescence intensity		↑	↓	↓		↓			
Fluorescence lifetime	*τ*_mean_	↓				↑	↓		
	*τ*_1_	↓				↓			
	*τ*_2_	↑		↓		↓	↑		
	α _1_	↑		↓	↑	↓	↑	↓	

summarizes changes observed in the autofluorescence intensity and lifetime characteristics of cardiac tissue due to oxygen or glucose depletion.

Prior to performing measurements using the column-switching configuration presented here, we performed autofluorescence measurements during ischemia in the Langendorff model (data not shown). Global ischemia was induced by stopping the flow of perfusate to the aorta which, as well as removing the supply of oxygen and glucose, removes flow of liquid to the heart. In our first experiment we observed a ~300 ps (n = 5) increase in fluorescence lifetime in channel 2, however we realized subsequently that this was at least partly due to the decrease in the temperature of the heart from 37.5°C down to 22.5°C when the flow of the warming perfusate stopped. In a separate study (data not shown), we measured an increase in the intensity-weighted mean lifetimes of free NADH, mMDH-bound NADH and LDH-bound NADH in aqueous solution of ~60, ~120 and ~300 ps respectively when the temperature is changed from 37 to 25°C. Therefore, these temperature dependencies could easily account for the increase in fluorescence lifetime seen. We then tried to reduce the change in temperature of the heart when flow is stopped by adding a heating cup that surrounded the heart from all sides but the top, but we still observed a decrease in temperature down to 28°C (n = 1). Finally, we attempted physically immersing the heart in a bath of warmed perfusate but this was difficult physically and we were still not able to remove changes in temperature when flow of perfusate to aorta was stopped. This preliminary work led us to the column-switching configuration used for the experiments presented in this paper, where changes in temperature on switching are minimized and the fluorescence lifetimes reported in the control experiments remain comparable before and after the column switch.

## 5. Conclusions

In this investigation we used a Langendorff-perfused isolated rat heart model to characterize the autofluorescence signatures of cardiac tissue that accompany the progressive impairment of myocardial function due to oxygen or glucose depletion. We were able to characterize changes in the autofluorescence parameters associated with NAD(P)H and FAD, which are indicative of the metabolic state of the tissue. In general, our results demonstrate that both NAD(P)H and FAD autofluorescence intensity and decay parameters are sensitive to metabolic changes caused by oxygen depletion. On reperfusion, we also observed changes in NAD(P)H fluorescence intensity and FAD fluorescence decay properties relative to baseline. This may provide some information on either permanent damage to the tissue caused by oxygen depletion or on changes associated with the tissue recovering from oxygen depletion. Further research is needed to link the changes in autofluorescence observed to specific biochemical processes. Changes in cardiac tissue resulting from glucose-depletion are most clearly visible in autofluorescence parameters associated with NAD(P)H. We believe that the results presented in this study can establish a baseline that may aid the interpretation of future autofluorescence measurements of cardiac tissue.
